# Prevalence and risk factors of compassion fatigue in gynecological and obstetric nurses: a systematic review and meta-analysis

**DOI:** 10.3389/fpubh.2025.1590995

**Published:** 2025-07-14

**Authors:** Jia Lv, Xin-Yi Che, Ling-Ling Xie, Min Ren, Tian-Zhi Guo, Su-Hua Tu

**Affiliations:** ^1^School of Nursing, Southwest Medical University, Luzhou, Sichuan, China; ^2^Department of Obstetrics and Gynecology, Affiliated Hospital of Southwest Medical University, Luzhou, China

**Keywords:** nurse, obstetrics and gynecology, compassion fatigue, influencing factors, systematic review

## Abstract

**Objective:**

This study systematically evaluated the incidence and influencing factors of compassion fatigue (CF) among obstetric and gynecological nurses.

**Methods:**

Retrieve the cross-sectional study on the current occurrence status of CF among obstetric and gynecological nurses in the Web of Science, PubMed, Embase, Cochrane Library, CINAHL, CBM, CNKI, VIP and WANFANG database. Database were searched from the database establishment to September 8, 2024. Literature screening, data extraction and bias risk assessment were performed independently by two researchers. Stata17.0 software was used for meta-analysis.

**Results:**

A total of 15 studies involving 6,799 obstetrics and gynecology nurses were included in the analysis. The meta-analysis results indicated that the incidence of moderate to severe CF among these nurses was 77.6% (ES = 0.776, 95% CI: 0.725–0.827). Additionally, the scores for compassion satisfaction, burnout, and secondary trauma were 29.835 (95% CI: 27.703–31.967), 28.116 (95% CI: 25.458–30.775), and 25.021 (95% CI: 21.681–28.360), respectively, all exceeding the critical threshold, indicating a high risk of CF in obstetrics and gynecology nurses. The identified influencing factors for CF included exposure to traumatic events, number of children, social support, sleep quality, health status, years of service, and hospital region and grade.

**Conclusion:**

The prevalence of CF among obstetrics and gynecology nurses is notably high, necessitating the implementation of individualized intervention strategies based on the identified influencing factors.

## 1 Introduction

### 1.1 Conceptual background

Compassion fatigue (CF) negatively impacts physical and mental health due to prolonged caregiving. This occurs when excessive emotional investment or exposure to recipients’ negative emotions overwhelms caregivers (1). This condition weakens the capacity for empathy and is often referred to as the “cost of care,” which adversely affects both psychological and physical well-being. The term “compassion fatigue” was first introduced by Joinson in 1992 (2), describing it as “a unique form of burnout that affects professionals in the nursing industry.” This condition can hinder the ability to sustain compassion and contribute to the erosion of nurses’ mental health. Consequently, some nurses may view leaving the profession as the only means of coping, making CF a significant factor in the global nursing shortage (3).

### 1.2 Current situaton of compassion fatigue

Research conducted by Oktay and Ozturk (4) indicates that nearly all nurses have experienced varying degrees of CF, which can diminish their physical and mental health, reduce work efficiency, and potentially lower patient satisfaction across physiological, social, emotional, spiritual, and cognitive dimensions (5).

### 1.3 Gender differences

Studies have indicated that women experience higher levels of CF compared to men (2), and since the majority of gynecological nurses are women, they are particularly susceptible to this phenomenon.

### 1.4 Policy context

In recent years, the comprehensive liberalization of the “two-child” and “three-child” policies, along with advancements in assisted reproductive technology, has led to a rising incidence of pregnancy complications and an increasing proportion of high-risk pregnant women (6). Gynecological nurses frequently encounter stress in their clinical roles, which can lead to CF, particularly among female nurses (7). Moreover, as a group that actively responds to the needs of second-time mothers, maternity leave and breastfeeding leave—both legally mandated—inevitably impact the availability of nursing staff in obstetrics (8). Additionally, obstetric nurses operating in a context of low fertility may experience heightened stress due to concerns about their future career development.

### 1.5 Measurement tools

In 1995, Figley proposed a two-factor theory, dividing CF into the dimensions of burnout and secondary trauma (9). Subsequently, Stamm (10) in the United States introduced a three-factor model of CF, adding “compassion satisfaction” to the original dimensions of burnout and secondary trauma, thereby enhancing the understanding of the concept. In 2005, Stamm developed the Professional Quality of Life Scale (ProQOL), which encompasses three scoring dimensions: compassion satisfaction, burnout, and secondary trauma. This scale has become the preferred tool for evaluating CF internationally (11) and was introduced to China in 2011. The ProQOL-V scale was first translated into traditional Chinese characters by Taiwanese scholars. In 2013, Zheng et al. (12) translated and adjusted the ProQOL scale for application in the context of professional quality of life among nurses, revising it to align with Chinese language norms in simplified characters. This adaptation has facilitated research on CF within the Chinese Nurses’ Association.

### 1.6 Study rationale

This study systematically evaluates the incidence and influencing factors of CF among gynecological nurses, explores the prevalent characteristics of the three key dimensions of compassion satisfaction, burnout, and secondary trauma, and provides a scientific reference for the formulation of intervention measures targeting CF in obstetrics and gynecology nursing.

## 2 Methods

### 2.1 Inclusion and exclusion criteria

*Inclusion criteria:* ① Cross-sectional studies; ② Chinese and English study published before September 24, 2024; ③ Subjects in study must be registered nurses in obstetrics and gynecology with over 6 months of clinical work experience; ④ The study variable is compassion fatigue; ⑤ Outcome indicators are measured using the ProQOL scale, which encompasses three dimensions of CF: compassion satisfaction, burnout, and secondary trauma. Additionally, the outcome indicators include related influencing factors or the incidence of moderate or severe CF.

*Exclusion criteria:* ① Duplicate publications or literature without available full text; ② Reviews and other secondary studies; ③ Documents with inconsistent data before and after the same document, or relevant data that cannot be extracted or transformed; ④ Literature with a low quality evaluation level; ⑤ Literature with a high risk of bias (bias risk score ≤3).

### 2.2 Literature search strategy

This study was conducted through electronic database searches on Web of Science, PubMed, Embase, Cochrane Library, CINAHL, China Biomedical Literature Database (CBM), CNKI, Wanfang, VIP and other Chinese and English databases to collect literature on the incidence of CF and related risk factors among obstetrics and gynecology nurses. The time limit of the search is established until September 8, 2024, and the search adopts the method of combining subject words and free words, and the search method is adjusted according to the characteristics of each database. With “nurs*” OR “compassion fatigue” OR “secondary trauma*” OR “vicarious trauma*”; AND “obstetric*” OR “gynecolog*” OR “midwi*” are indexed to search English databases; The references included in the study were retrospectively retrieved through manual retrieval of “nurse” or “obstetrics” or “gynecology” or “midwife” or “obstetrical medical staff” and “compassion fatigue” or “CF” to obtain additional relevant literature and improve the recall rate.

### 2.3 Literature screening and data extraction

Two independent researchers screened the literature independently, following the research objectives and exclusion criteria. They extracted relevant information, cross-checked their findings, and consulted with a third researcher to resolve any discrepancies. During the literature selection process, the title of each work was reviewed first, followed by an examination of the abstract and full text to determine inclusion after excluding obviously irrelevant studies. Authors of the original studies were contacted via email or phone to obtain any uncertain but crucial information regarding the studies. Data were extracted from the included literature using a pre-prepared data extraction table, which will encompassed: ① Basic information about the included studies, such as the first author, publication year, country, sample size, research subjects, survey tools, and incidence rates; ② Basic information about the nurses, including sample size and demographic data; ③ Scores related to compassion satisfaction, burnout, and secondary trauma; ④ Main influencing factors of CF.

### 2.4 Literature bias risk assessment

Two researchers will independently evaluated the risk of bias in the included studies and subsequently compared their results. In the event of any disagreement, a third researcher was consulted for resolution. The bias risk assessment utilized the cross-sectional research evaluation criteria recommended by the Agency for Healthcare Research and Quality (AHRQ) in the United States (13), which comprises 11 indicators. Each study will be scored with responses of “yes,” “no,” or “unclear,” where “yes” receives 1 point, while “no” or “unclear” receives 0 points. Research quality will be categorized into three grades based on the total score: a score of ≤ 3 will be classified as low-quality research, 4–7 as medium-quality research, and 8–11 as high-quality research.

### 2.5 Statistical methods

Zotero literature management software was employed to organize and summarize the literature. Excel software was utilized for data extraction management, as well as for statistical and descriptive analysis of outcome indicators. Stata 17.0 software was used to conduct meta-analysis of rates, employing the metan command. The Q test assessed the heterogeneity among the results of the included studies, with a significance level set at *α* = 0.1 for this evaluation. This relatively lenient threshold (*α* = 0.1) for heterogeneity detection aims to maximize sensitivity in identifying potential between-study variability, while the stricter α = 0.05 threshold for overall effect significance maintains appropriate Type I error control. Heterogeneity will be quantitatively assessed; if *p* > 0.1 and *I^2^* ≤ 50%, the heterogeneity among studies will be regarded as acceptable, and a fixed-effect model will be applied for meta-analysis. Conversely, if substantial statistical heterogeneity is observed (*p* ≤ 0.1, I^2^ > 50%), a random effects model will be utilized, because random effects models include random effects terms to capture unobserved group differences in data, they better identify heterogeneity sources and improve estimate reliability. The significance level for the meta-analysis will be established at *α* = 0.05. Since≥ 10 studies were included, Publication bias will be assessed using Egger’s test and funnel plots, with *p* > 0.05 indicating no significant publication bias. Given the variations in the definitions of influencing factors across the included studies, qualitative analysis will be conducted to analyze and summarize these factors. *p* < 0.05 indicated that the difference in statistical results was statistically significant.

## 3 Results

### 3.1 Literature screening process and results

**Figure 1:**
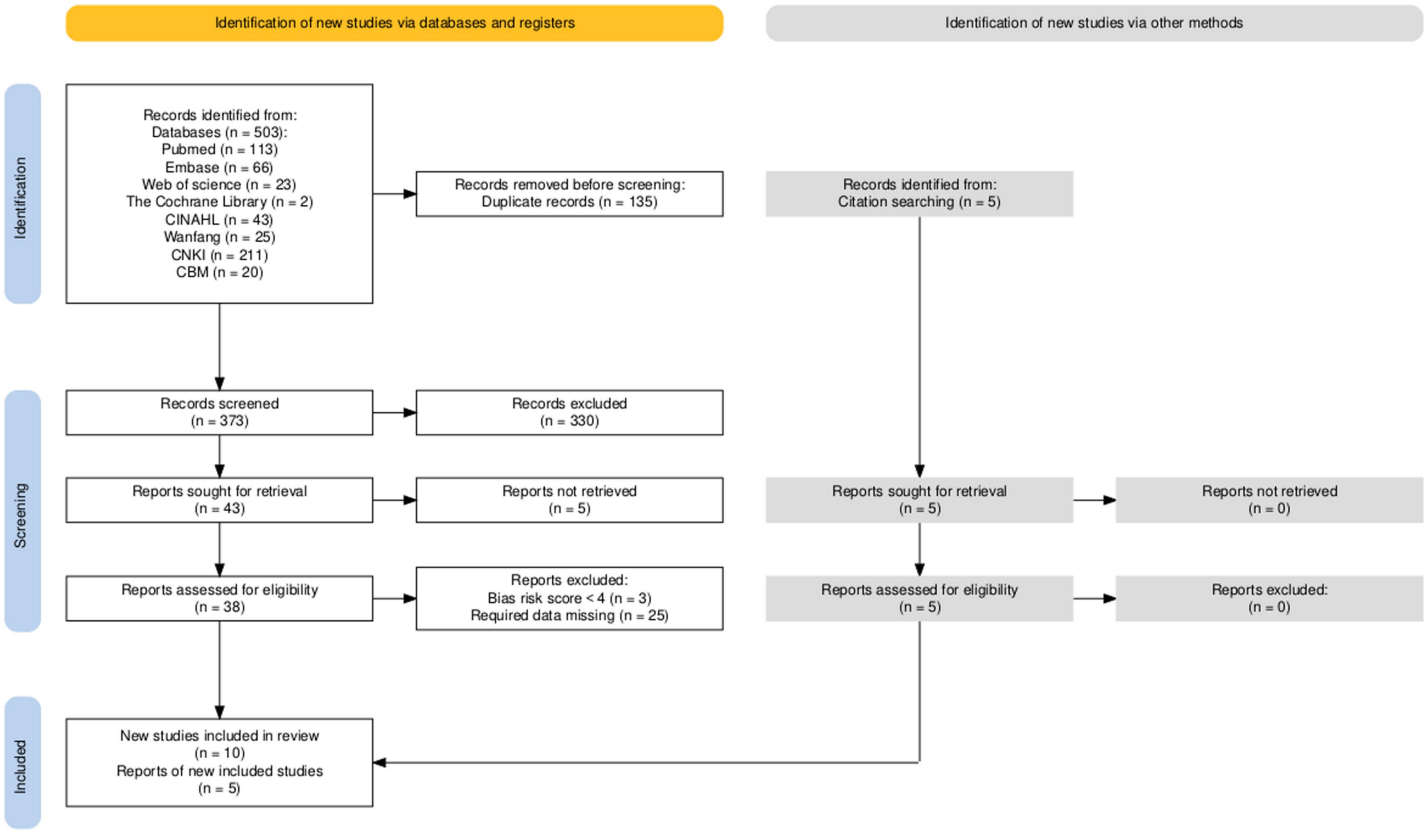
PRISMA flow diagram.

A total of 507 literature sources were obtained during the preliminary search, including four additional sources sourced from other resources. After reviewing the literature, 327 sources were selected based on various themes, research objects, research types, and the removal of duplicates, resulting in 181 sources remaining. Following an evaluation of the titles, abstracts, and keywords, 138 sources were excluded, leading to a final review of the full text of 43 sources. From these, 28 sources were eliminated, and ultimately, 15 (14–28) cross-sectional studies were selected for inclusion in the analysis, as illustrated in Figure 1

**Figure 2:**
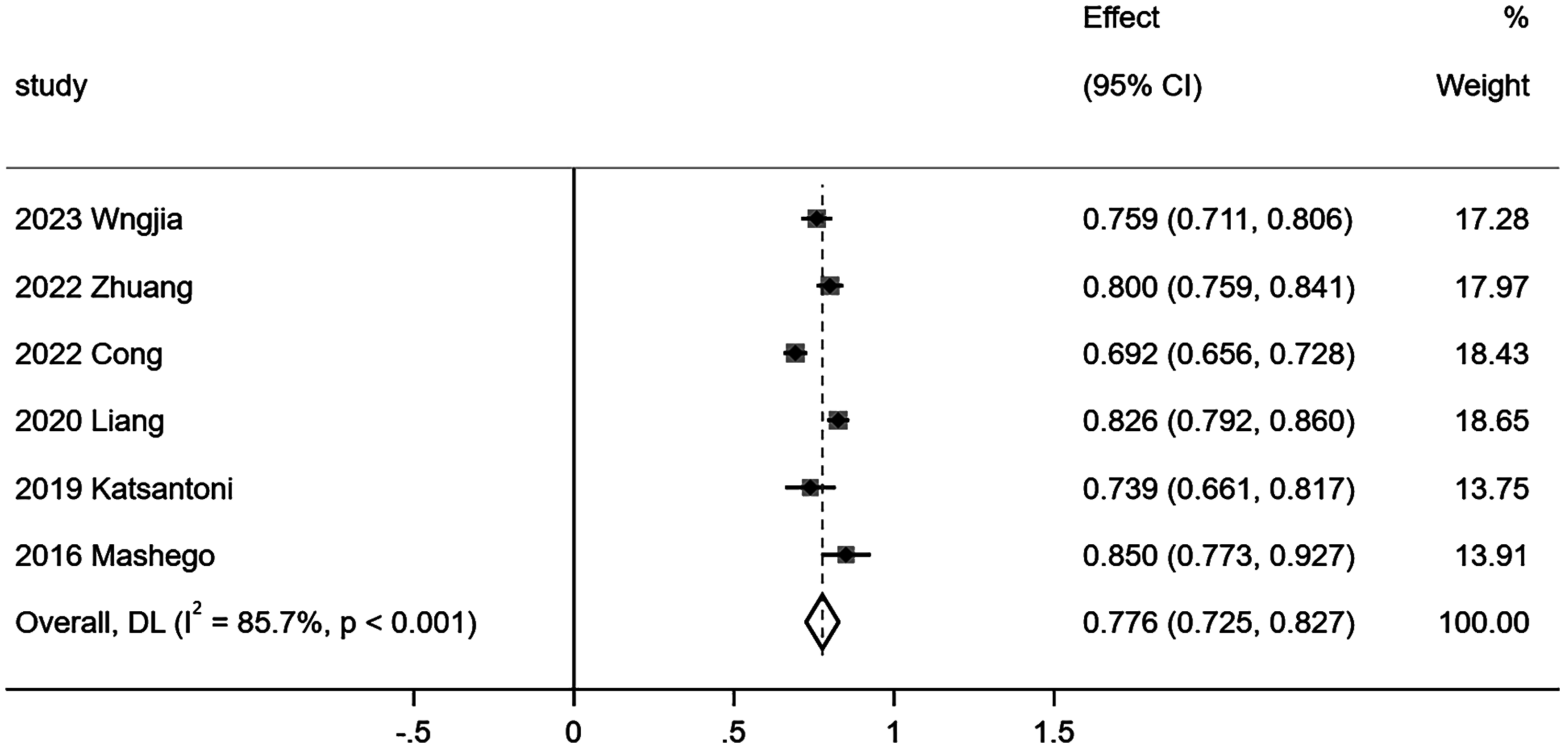
Forest chart of incidence of moderate and severe CF in gynecological nurses.

### 3.2 Basic features of included studies

A total of 15 articles (14–28) (6 in Chinese and 9 in English) were included in this systematic review, encompassing a total of 6,799 gynecological nurses. The fundamental characteristics of the included studies are presented in Table 1.

**Table 1:** Basic features of included studies.

Author name	Year	Country	Assessment tool	Object of study	Sample size	Moderate–severe CF incidence	Influencing factor
Alice (14)	2024	Italy	ProQOL	Midwives	286	NA	Work trauma
Wang Jia (15)	2023	China	ProQOL	Obstetric and gynecological nurses	311	75.88%	Physical condition, number of children, emotional labor, lack of professional effectiveness, non-only child, social support, night shift, work experience
Aydin (16)	2023	Turkey	ProQOL	Midwives	3,100	NA	Age, shift work (wheel white. Night), number of traumatic births, years worked
Qu Lixia (17)	2022	China	ProQOL	Midwives	206	NA	Personality, health status, job satisfaction, shift status, sleep quality, exposure to traumatic birth events, average weekly work hours, social support, major changes, job satisfaction, job recognition, work load
Zhuang (18)	2022	China	ProQOL	Midwives	365	80%	Age, marriage, number of preschool children, number of traumatic events experienced during childbirth, job satisfaction, sleep quality, excessive work load, and harmonious work atmosphere
Cong (19)	2022	China	ProQOL	Obstetric and gynecological nurses	618	69.2%	Years of work, children, social support, psychological capital, job title
Liang (20)	2020	China	ProQOL	Midwives	477	82.6%	Hospital level, child status, region, preferred working atmosphere, number of birth traumatic events, sleep quality, degree of social support
Dong (21)	2020	China	ProQOL	Obstetric nurses	188	NA	Positive psychological quality
Katsantoni (22)	2019	Greece	ProQOL	Obstetric and gynecological nurses	121	73.9%	NA;
Huang (23)	2019	China	ProQOL	Obstetric and gynecological nurses	229	NA	Age, marriage, professional title, educational background, career experience, career happiness
Feng (24)	2019	China	ProQOL	Obstetric and gynecological nurses	243	NA	Professional identity
Cohen (25)	2017	Israel	ProQOL	Midwives	93	NA	Traumatic birth events
Muliira (26)	2016	Uganda	ProQOL	Midwives	224	NA	Age, education, working years, working area, health status
Mashego (27)	2016	South Africa	ProQOL	Obstetric nurses	83	85%	Experience a traumatic birth event
Mizuno (28)	2013	Japan	ProQOL	Obstetric nurses and midwives	255	NA	Experiencing traumatic birth events, emotions

NA, not available.

### 3.3 Bias risk evaluation of included studies

This summary encompasses eight (15, 18–21, 23, 24, 26) high-quality studies and seven (14, 16, 17, 22, 25, 27, 28) medium-quality studies, with an assessment of the risk of bias for each. Specific results can be found in Table 2.

**Table 2:** Results of bias risk assessment for included studies.

Included studies	Entries											Score (points)	Quality level
	1	2	3	4	5	6	7	8	9	10	11		
2024 Alice (14)	Yes	Yes	Yes	Yes	Unclear	Yes	No	Yes	Unclear	Yes	No	7	Medium
2023 Wang Jia (15)	Yes	Yes	Yes	Yes	Unclear	Yes	Yes	Yes	Yes	Yes	No	9	High
2023 Aydin (16)	Yes	Yes	Yes	Yes	Unclear	Yes	No	Unclear	No	Yes	No	6	Medium
2022 Qu Lixia (17)	Yes	Yes	Yes	Yes	Unclear	Yes	Yes	Unclear	No	Yes	No	7	Medium
2022 Zhuang (18)	Yes	Yes	Yes	Yes	Unclear	Yes	Yes	Unclear	Yes	Yes	No	8	High
2022 Cong (19)	Yes	Yes	Yes	Yes	Unclear	Yes	Yes	Yes	No	Yes	No	8	High
2020 Liang (20)	Yes	Yes	Yes	Yes	Unclear	Yes	Yes	Yes	Yes	Yes	No	9	High
2020 Dong (21)	Yes	Yes	Yes	Yes	Unclear	Yes	Yes	Unclear	Yes	Yes	No	8	High
2019 Katsantoni (22)	Yes	Yes	Yes	Yes	Unclear	Yes	No	Unclear	No	Yes	No	6	Medium
2019 Huang (23)	Yes	Yes	Yes	Yes	Unclear	Yes	Yes	Unclear	Yes	Yes	No	8	High
2019 Feng (24)	Yes	Yes	Yes	Yes	Unclear	Yes	Yes	Yes	No	Yes	No	8	High
2017 Cohen (25)	Yes	Yes	Yes	Yes	Unclear	Yes	No	Unclear	Yes	Yes	No	7	Medium
2016 Muliira (26)	Yes	Yes	Yes	Yes	Unclear	Yes	Yes	Yes	No	Yes	No	8	High
2016 Mashego (27)	Yes	Yes	Yes	Yes	Unclear	Yes	No	Unclear	No	Yes	No	6	Medium
2013 Mizuno (28)	Yes	Yes	Yes	Yes	Unclear	Yes	Yes	Unclear	No	Yes	No	7	Medium

Item 1 is whether the data source is clear (investigation, review of medical record data), item 2 is whether the exclusion/inclusion criteria for exposed and non-exposed groups (trial group or control group) are clear or whether reference is made to previous publications, item 3 is whether the time period of patients is clear, and item 4 is whether the continuity of subjects is clear if it is not a population-based study. Article 5 is whether other aspects of the subjects were influenced by the evaluator’s subjective factors, article 6 is whether any evaluation measures for quality assurance (such as testing/retesting of the primary outcome) are described, article 7 is whether the reasons for excluding any patients from the analysis are explained, and article 8 is whether the methods for evaluating and/or controlling confounding factors are described. Entry 9 explains how to deal with lost data from the analysis, if possible, entry 10 summarizes patient response rates and the integrity of data collection, and entry 11, if possible, describes expected follow-up and the percentage of incomplete data or follow-up obtained.

### 3.4 Results of meta-analysis

#### 3.4.1 Risk of compassion fatigue

**Figure 3:**
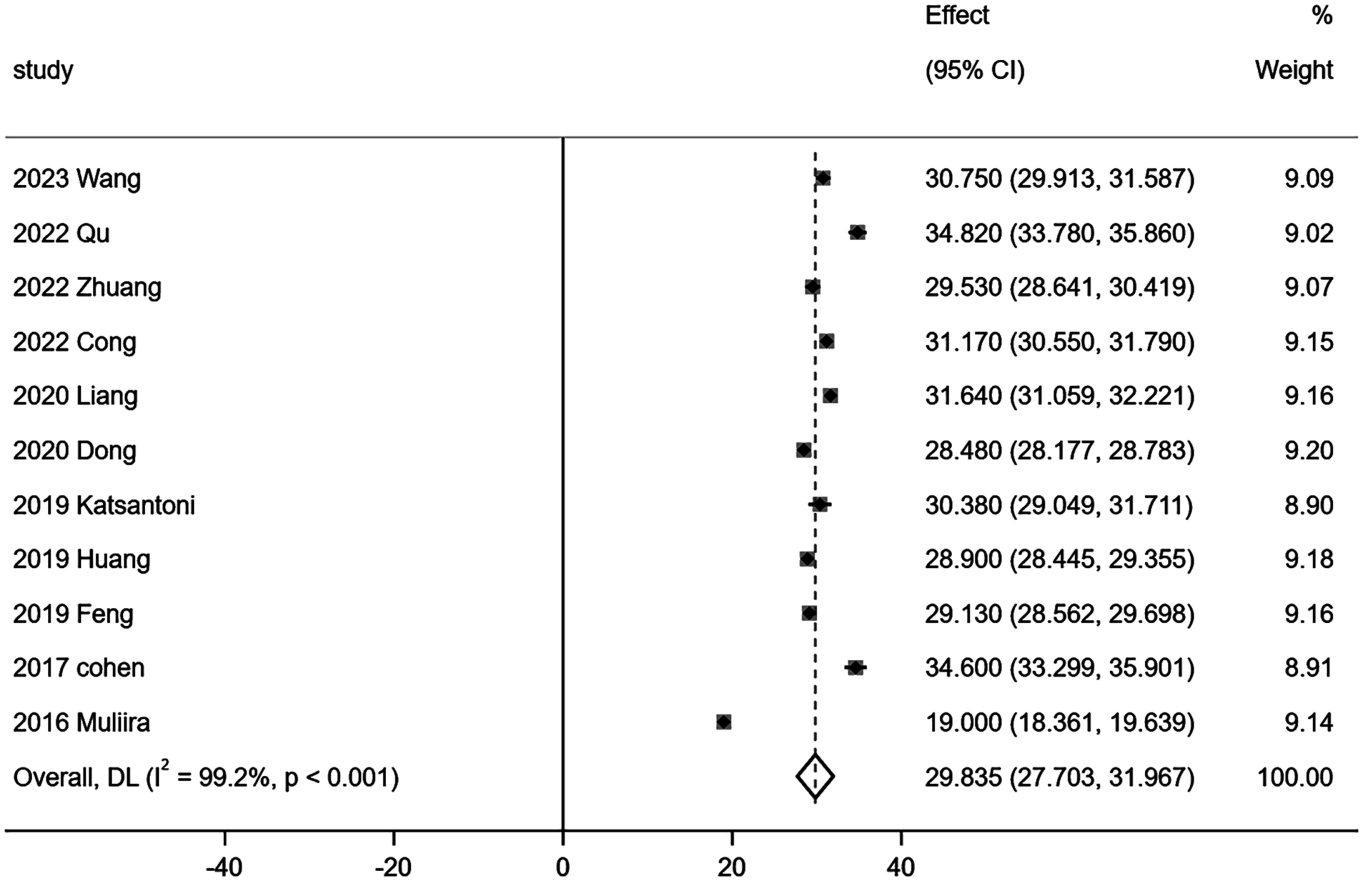
Forest map of compassion satisfaction score.

**Figure 4:**
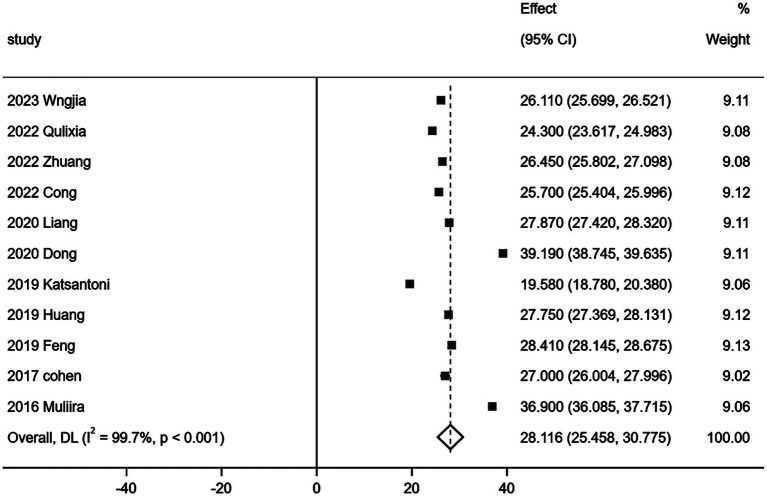
Forest map of burnout score.

**Figure 5:**
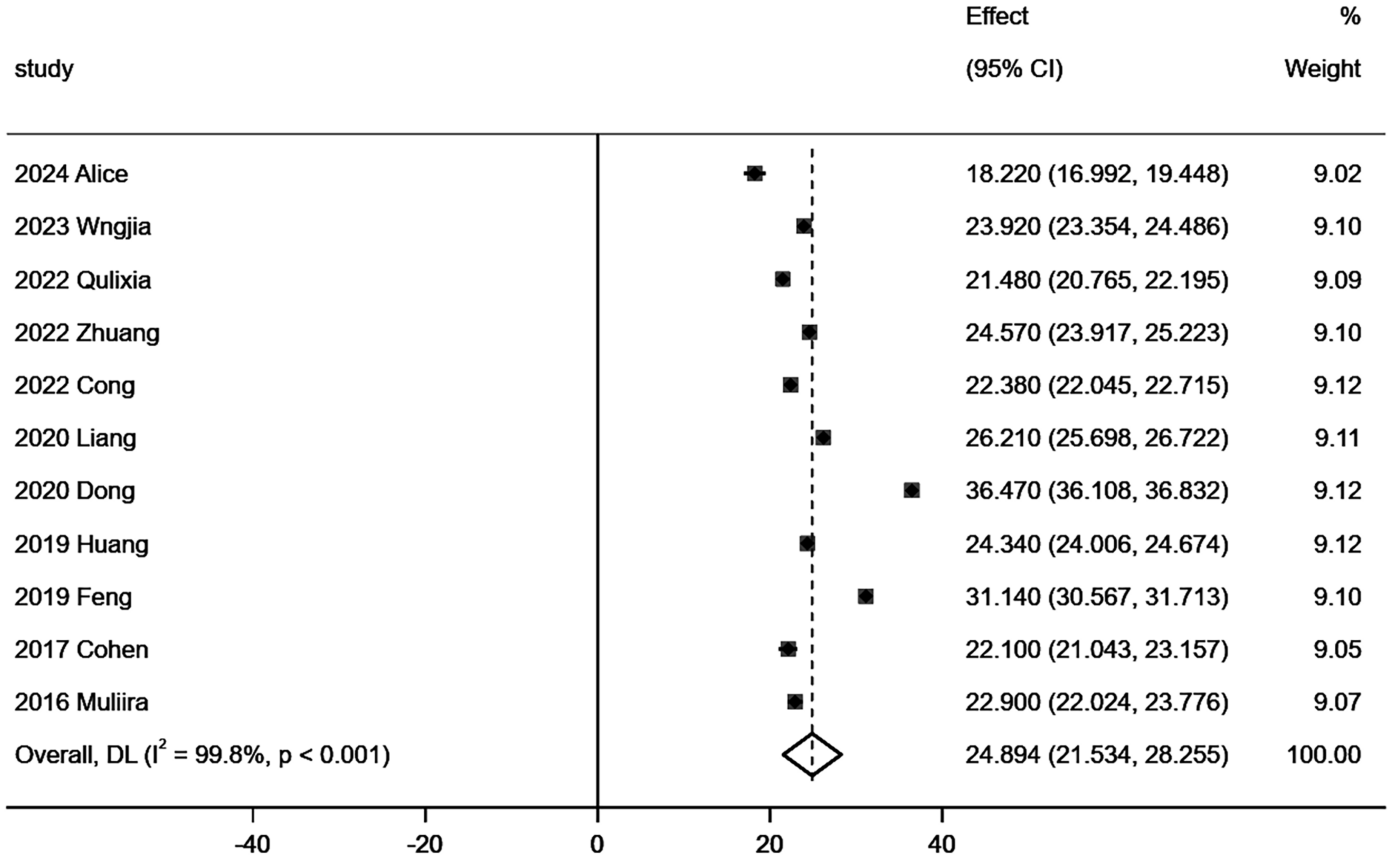
Forest map of secondary trauma score.

Eleven (15, 17–26) out of 15 studies comprehensively reported the three dimensions of CF as measured by the Professional Quality of Life Scale (ProQOL). The relevant data are presented in Table 3. Given the considerable heterogeneity observed in the quantitative evaluation results of the three ProQOL dimensions across these studies, a random effects model was employed for the meta-analysis of the 11 studies (*n* = 3,075). The results of this analysis are illustrated in Figures 3–5.

**Table 3:** ProQOL scores in each dimension.

Study	Sample size	ProQOL score		
		Compassion satisfaction (CS)	Burnout (BO)	Secondary trauma score (STS)
2023 Wang Jia (15)	311	30.75 ± 7.53	26.11 ± 3.70	23.92 ± 5.09
2022 Qu Lixia (17)	206	34.82 ± 7.62	24.30 ± 5.00	21.48 ± 5.24
2022 Zhuang (18)	365	29.53 ± 8.67	26.45 ± 6.32	24.57 ± 6.37
2022 Cong (19)	618	31.17 ± 7.86	25.70 ± 3.75	22.38 ± 4.25
2020 Liang (20)	477	31.64 ± 6.47	27.87 ± 5.01	26.21 ± 5.70
2020 Dong (21)	188	28.48 ± 2.12	39.19 ± 3.11	36.47 ± 2.53
2019 Katsantoni (22)	121	30.38 ± 7.47	19.58 ± 4.49	19.61 ± 7.45
2019 Huang (23)	229	28.90 ± 3.51	27.75 ± 2.94	24.34 ± 2.58
2019 Feng (24)	243	29.13 ± 4.52	28.41 ± 2.11	31.14 ± 4.56
2017 Cohen (25)	93	34.60 ± 6.40	27.00 ± 4.90	22.10 ± 5.20
2016 Muliira (26)	224	19.00 ± 4.88	36.90 ± 6.22	22.90 ± 6.69

#### 3.4.2 Sensitivity analysis

**Figure 6:**
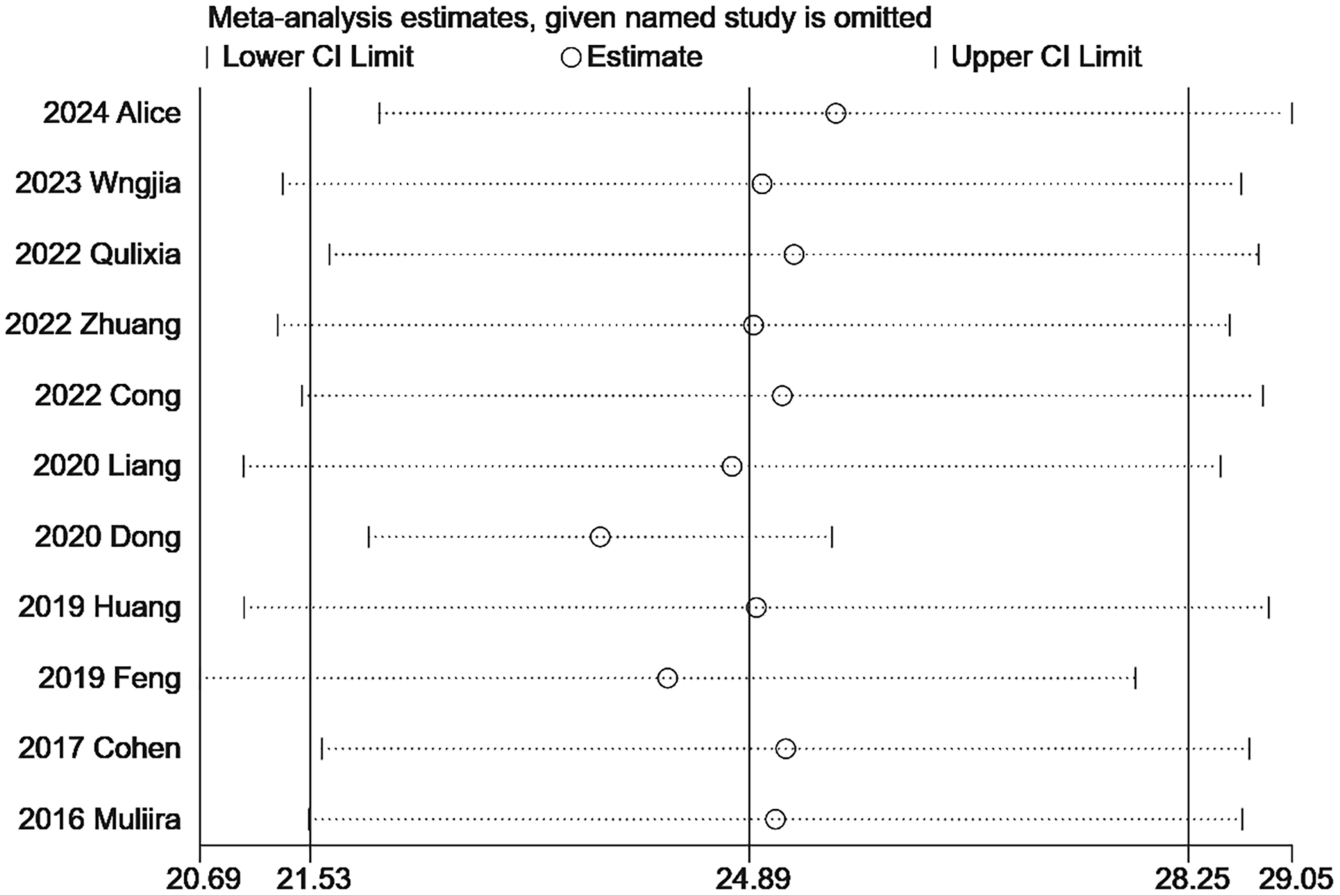
Sensitivity analysis of secondary trauma score of obstetrics and gynecology nurses.

Due to the high heterogeneity of the included studies, a sensitivity analysis is necessary. Following the inclusion of the studies, the results obtained did not exhibit significant changes in the combined and estimated values, indicating that the findings of this study are stable and reliable. The sensitivity analysis results concerning secondary trauma are presented in Figure 6. Additionally, the sensitivity analyses for compassion satisfaction and burnout further demonstrate the stability of these results.

#### 3.4.3 Publication bias

**Figure 7:**
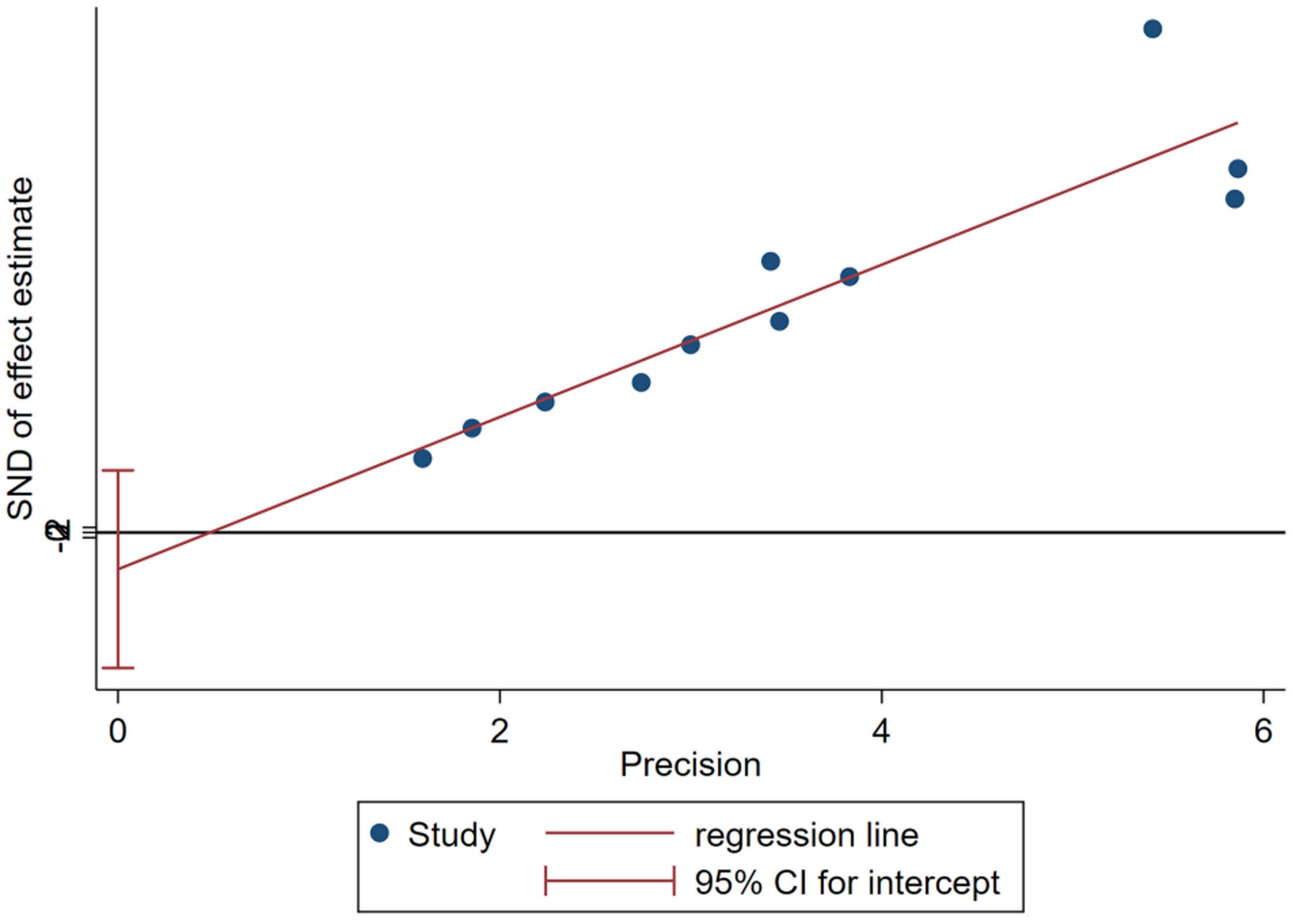
Funnel plot of secondary trauma score of gynecological nurses.

The Egger test was employed to quantitatively assess publication bias, yielding the following results: *p* = 0.443 for compassion satisfaction (*p* > 0.05); *p* = 0.951 for burnout (*p* > 0.05); and *p* = 0.422 for secondary trauma (*p* > 0.05). The funnel plot is illustrated in Figure 7. These findings suggest that the literature included is extensive and comprehensive, indicating a low likelihood of publication bias.

## 4 Discussion

### 4.1 Gynecological nurses with high risk of compassion fatigue

This study comprehensively analyzed the relevant Chinese and English literature published before September 24, 2024, Meta analysis was conducted on the literature that fully reported three dimensions of CF in the included literature, and the results showed that there were three dimensions of CF: compassion satisfaction, burnout and secondary trauma. Their scores were 29.835 [95%*CI* (27.703, 31.967)], 28.116 [95%*CI* (25.458, 30.775)] and 25.021 [95%*CI* (21.681, 28.360)], respectively. All three dimensions—compassion satisfaction, burnout, and secondary trauma—exceeded the critical values. According to the scale’s usage guidelines, this suggests that the risk of CF among obstetrics and gynecology nurses is classified as severe. The critical values for the scale are set at 27 points for mild CF and >17 points for moderate CF. If one dimension’s score exceeds the critical value, it indicates mild CF; if two dimensions exceed, it indicates moderate CF; and if the total score of all three dimensions exceeds the critical value, it is considered severe CF (12). Furthermore, the systematic evaluation revealed that the incidence of moderate and severe CF among nurses in obstetrics and gynecology was 77.6% [*ES* = 0.776, 95% *CI* (0.725, 0.827)].

#### 4.4.1 Incidence of CF

Among the included literatures, six (15, 18–20, 22, 27) literatures reported the incidence of CF in obstetrics and gynecology nurses. Due to multiple studies, the incidence of normal and mild fatigue was combined (15, 19), and the incidence of moderate and severe CF was combined (20, 22, 27). Therefore, the incidence rate of moderate and severe CF was analyzed in this study, and heterogeneity test was carried out on the included studies (*I^2^* = 85.7%, *p* < 0.001), indicating that there was a high degree of heterogeneity among the included studies. Random-effects model was used to combine the effect size of each study, *ES* = 0.776, 95%*CI* (0.725–0.827). It is suggested that the incidence of violence is about 77.6%, as shown in Figure 2. Due to the small number of studies included in the meta-analysis of incidence, funnel plots were not drawn.

### 4.2 Analysis of the influencing factors of compassion fatigue among gynecological nurses in obstetrics and gynecology

The findings indicate that an increased number of traumatic events, a higher number of children, reduced social support, poorer sleep quality, and less healthy nurses are associated with an elevated risk of CF. (1) Specifically, the frequency of traumatic events experienced correlates positively with the risk of CF. Unlike other departments, maternal and family members often struggle to anticipate potential adverse outcomes and lack adequate psychological preparation. Consequently, during emergencies, they are frequently unable to cope, which may lead to emotional distress or extreme reactions. Additionally, these emergencies can inflict psychological trauma and frustration on nurses (20), thereby increasing their vulnerability to CF (17, 22). For nursing managers, it is crucial to establish a non-punitive management system for errors and adverse events in the context of traumatic occurrences, while also addressing the psychological well-being of nurses. Implementing tiered skill training strategies to enhance nurses’ capabilities in managing urgent and critical emergencies is a vital measure to mitigate the impact of traumatic childbirth (20). (2) Furthermore, considering the number of children, it is noteworthy that 87.5% of obstetrics and gynecology nurses are in early motherhood, navigating multiple roles such as mothers, daughters, and wives. This multifaceted responsibility can lead to conflicts between work and family, especially when caring for multiple dependents (15). Nurses with more than two preschool children, particularly those with infants and young children at home, are at an increased risk of experiencing CF due to the heightened demands of their parental roles (18, 20). (3) A lower level of social support correlates with a higher risk of CF. Insufficient support from colleagues and leaders at work, along with a lack of understanding from family and friends in personal life, can create dual pressures from both work and home (17). Consequently, the establishment of a comprehensive support system requires the collaboration and efforts of individuals, families, friends, and the broader society. Nurses must navigate their changing roles and strive to achieve a better balance between work and personal life. Nursing managers should encourage families and colleagues to provide assistance, guidance, and support, while also ensuring that job responsibilities are arranged reasonably and tasks are planned effectively. This will help guarantee that nurses have adequate rest time, facilitating their transition between multiple roles (19). (4) Additionally, nurses in poor health face an increased risk of CF. Physical health is a fundamental source of energy; when health declines, the balance of resources is disrupted, leading to diminished compassion. In severe cases, this can result in the onset of CF (15, 17). (5) From the perspective of sleep quality, inadequate sleep not only contributes to the development of CF but also manifests as a physical symptom of it (20). Furthermore, sleep quality can significantly impact overall health status. Therefore, it is recommended that obstetric nurses prioritize maintaining a healthy lifestyle and adequate rest, undergo regular physical examinations, and ensure their physical and mental well-being.

However, there are still differences in the study results regarding whether factors such as age, years of experience, shift mode, hospital area, and departmental grade of obstetrics nurses influence the degree of CF. (1) Age: Studies have shown that as age increases, obstetrics nurses’ scores for sympathy fatigue also rise, indicating an increase in CF (16, 18). Further research has explored the relationship between empathy and age among obstetric nurses (15, 20). (2) Years of experience: Research by Wang et al. (15) indicates that nurses with both shorter and longer working years (over 16 years) exhibit lower levels of CF. It is posited that less experienced nurses may feel less familial responsibility and possess stronger capabilities and mature thinking, while those with extensive experience might struggle to manage the high levels of CF resulting from balancing family and work responsibilities (29). Conversely, some studies suggest that longer working hours correlate with lower CF scores, potentially due to increased work proficiency (23). It is recommended that nursing managers adopt a nuanced perspective on years of experience. When assessing their impact on compassion satisfaction, it is crucial to consider the specific context, accurately identify high-risk individuals, and enhance their education and training to improve their capacity to assist others. Furthermore, particular attention should be given to the competitive nature of senior nurses’ professional titles, ensuring they receive appropriate professional guidance and support (19). (3) Shift Mode: Numerous studies have indicated that night work increases levels of sympathy and fatigue among obstetricians (15, 17). However, research has also demonstrated that while midwives who frequently work night shifts report higher scores of CF, there is no significant correlation between work style and CF (16). This phenomenon may be associated with the individual sleep habits of nurses, suggesting that nursing managers should implement flexible scheduling practices. (4) Hospital Areas and Grades: It is posited that midwives in remote, underserved areas or primary hospitals are more susceptible to experiencing CF due to factors such as limited resources, low salaries, and insufficient support (26). This situation is particularly evident in secondary hospitals across China and in western regions, where challenges related to nurse availability, medical conditions, and salary levels are pronounced. Specifically, in western China, there is a high demand for nurses, coupled with inadequate emergency and critical care capabilities, limited training opportunities, and a significant need for specialized training. These issues disrupt the equilibrium of health resource distribution. Conversely, another perspective suggests that nurses in tertiary hospitals experience higher levels of CF, potentially linked to the rising number of older adult and high-risk women following the adjustment of China’s reproductive policy. This demographic shift has led to increased urgency and severity in rescue efforts, elevating work risks in comprehensive tertiary hospitals. To address these challenges, it is essential to strengthen the development of grassroots medical and health professionals in alignment with policy direction, promote collaborative mechanisms among medical institutions within healthcare associations (30), and implement the “corresponding assistance” plan from tertiary hospitals to secondary hospitals. Such measures aim to optimize the allocation of medical resources and foster balanced development across hospitals and regions at all levels (20).

In summary, obstetrics and gynecology nurses experience a significant level of CF and should prioritize self-psychological care in their daily practice. Nursing managers need to be particularly attentive to obstetrics and gynecology nurses who are frequently exposed to traumatic events, as well as those who are managing higher workloads, inadequate sleep, and poor health. To safeguard the physical and mental well-being of nurses, it is essential to optimize obstetrics and gynecology nursing staffing, develop more reasonable shift arrangements, ensure adequate rest periods, and alleviate work-related stress. Additionally, providing support for obstetrics and gynecology nurses with heavy family responsibilities, fostering a positive working atmosphere, and enhancing social support can lead to greater job satisfaction and overall happiness among nurses. Furthermore, integrating trauma management techniques with emotional literacy training can effectively address post-traumatic stress and mitigate the risk of CF.

## 5 Limitations and prospects

Due to language limitations, only literature in Chinese and English was included in the search, which may impact the comprehensiveness of the research findings. The included studies exhibit a high degree of heterogeneity, likely related to variations in study populations, geographic regions, and hospital management systems across different countries. Nevertheless, further sensitivity analyses indicated that the study’s results are reliable. Future research should expand the subgroup analysis to explore the three dimensions influencing CF among gynecology and obstetrics nurses. Currently, investigations into the factors influencing CF in these nurses remain insufficiently comprehensive, with many factors being evaluated inconsistently and limited supporting literature for certain risk factors. Consequently, more evidence is required to further establish causal relationships. It is essential to conduct additional high-quality studies to substantiate these findings. The CF experienced by nurses in obstetrics and gynecology not only impacts their physical and mental well-being but also affects their nursing efficiency and quality of care. Therefore, researchers should investigate relevant evidence based on modifiable influencing factors and develop supportive interventions aimed at reducing CF in obstetrics and gynecology nurses.
